# Systematic assessment of outcomes following a genetic diagnosis identified through a large-scale research study into developmental disorders

**DOI:** 10.1038/s41436-021-01110-3

**Published:** 2021-02-18

**Authors:** H. Copeland, E. Kivuva, H. V. Firth, C. F. Wright

**Affiliations:** 1 Peninsula Clinical Genetics, Royal Devon and Exeter NHS Foundation Trust, RD&E Heavitree, Exeter, UK; 2 Box 134 Addenbrooke’s Hospital, Cambridge University Hospitals, Cambridge, UK; 3 Institute of Biomedical and Clinical Science, University of Exeter Medical School, RILD Building, Royal Devon & Exeter Hospital, Exeter, UK

## Abstract

**Purpose:**

The clinical and psychosocial outcomes associated with receiving a genetic diagnosis for developmental disorders are wide-ranging but under-studied. We sought to investigate outcomes from a subset of families who received a diagnosis through the Deciphering Developmental Disorders (DDD) study.

**Methods:**

Individuals recruited through the Peninsula Clinical Genetics Service who received a confirmed genetic diagnosis through the DDD study before August 2019 (*n* = 112) were included in a clinical audit. Families with no identified clinical outcomes (*n* = 16) were invited to participate in semistructured telephone interviews.

**Results:**

Disease-specific treatment was identified for 7 probands (6%), while 48 probands (43%) were referred for further investigations or screening and 60 probands (54%) were recruited to further research. Just 5 families (4%) opted for prenatal testing in a subsequent pregnancy, reflecting the relatively advanced maternal age in our cohort, and 42 families (38%) were given disease-specific information or signposting to patient-specific resources such as support groups. Six interviews were performed (response rate = 47%) and thematic analysis identified four major themes: reaching a diagnosis, emotional impact, family implications, and practical issues.

**Conclusion:**

Our data demonstrate that receiving a genetic diagnosis has substantial positive medical and psychosocial outcomes for the majority of patients and their families.

## INTRODUCTION

Developmental disorders describe a group of conditions that result in delayed cognitive and/or atypical physical development in children under 5 years of age.^1^ While a wide range of etiologies are recognized, up to 80% of cases are predicted to be due to an underlying monogenic or chromosomal cause.^2,3^ Although standard clinical approaches are only able to identify diagnoses for less than half of affected individuals,^4^ family-based genome-wide sequencing studies have substantially increased the diagnostic yield over the last decade.^5,6^ The benefits of receiving a genetic diagnosis can be broad, from counseling the family about their recurrence risk and offering preimplantation/prenatal testing, through to identifying diagnosis-specific treatments for the proband.^7,8^ However, while a few recent studies have investigated the outcomes for families who received a clinical diagnosis through exome and genome sequencing, they mostly comprise case reports and small case series.^9^ To date, no research has systematically evaluated outcomes in a large cohort of patients who obtained their diagnosis through a genomic research study.

The Deciphering Developmental Disorders (DDD) study^4^ was created with the aim of using high-resolution genomic testing methods to investigate the genetic etiology of abnormal development. From 2011 to 2015, the study recruited ~13,500 children with severe but previously undiagnosed developmental disorders, and their parents, from 23 National Health Service (NHS) Regional Genetics Services across the UK and one in Eire.^10,11^ Using a combination of high-resolution array comparative genomic hybridization (array-CGH) testing and trio exome sequencing,^12,13^ the DDD study has identified plausible genetic diagnoses in around 40% of children and communicated these findings back to their local genetics teams for validation and discussion with the families.^11^ The DDD study thus provides an ideal cohort in which to systematically evaluate clinical outcomes arising from a pediatric genomics research study.

Here we describe a pilot study investigating the clinical and psychosocial outcomes of receiving a genetic diagnosis in a subset of 112 DDD participants from a single UK center. Our study will not only help better understand the wider impact of receiving a diagnosis for the patient and their family, but also inform larger systematic outcomes studies.

## MATERIALS AND METHODS

**Fig. 1:**
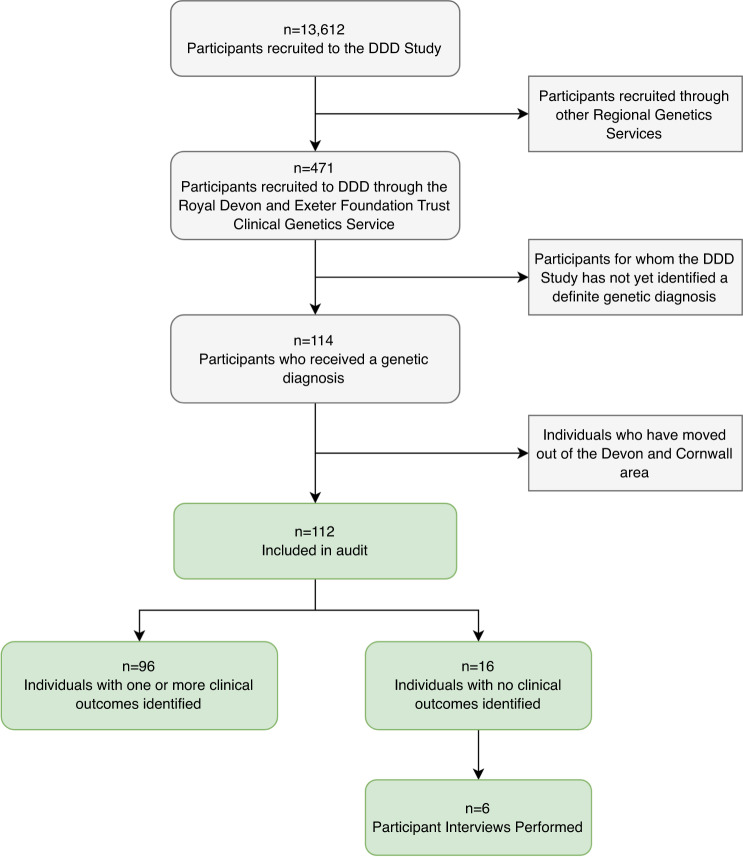
Flowchart showing recruitment process for the clinical audit and the semistructured interviews. DDD Deciphering Developmental Disorders study.

We used a mixed methods approach to gather outcome data on DDD families, which combined a quantitative clinical audit and a series of semistructured qualitative interviews. Eligibility for inclusion in the audit was defined as any individual recruited to the DDD study through the Peninsula Clinical Genetics Service (which covers Devon and Cornwall) for whom the DDD study had identified a definite genetic diagnosis (Fig. 1). Eligible individuals were identified through the DECIPHER database,^14^ and linked-anonymized DECIPHER IDs were used by the local team to connect the genetic diagnosis with the patient records for each proband. We developed a pro forma for collecting standardized information during the clinical audit from the local NHS Clinical Genetics records relating to outcomes; this included treatments, further investigations, reproductive decisions, adverse events, recruitment to further genetic research studies, and the information given to the patient/parents at the time of diagnosis (Supplementary Table 1). Disease-specific treatments were identified through review of correspondence in clinical records and through published literature searching. We also used the Unique patient support website^15^ and other online resources to identify whether gene disease–specific information was available, such as a leaflet or document written for patients and their families.

Following the clinical audit, families in whom we were unable to identify any outcomes were invited to participate in a semistructured interview. We designed a questionnaire (Supplementary Table 2) to identify personal, family, and wider outcomes that were not possible to assess through the audit. Interviews were performed over the telephone and recorded to enable transcription. A deductive thematic approach was used to analyze the qualitative data to identify major and minor themes.

## RESULTS

We identified 112 diagnosed probands who were eligible for inclusion in the clinical audit (Fig. 1). The probands ranged in age at recruitment from 3 to 44 years (median = 8.7), which is somewhat older than the rest of the cohort (median = 6.7, *p* = 0.0002), and the age of the parents at the birth of the proband ranged from 17 to 66 (median = 31), which is representative of the rest of the DDD cohort (median = 31, *p* = 0.3). A range of confirmed diagnoses were identified, including 97 dominant (of which 81 were de novo), 12 recessive, and 3 X-linked recessive. Within the cohort, variants were classed as pathogenic or likely pathogenic following the Association for Clinical Genomic Science (ACGS) best practice guidelines for variant classification^16^ and validated in an accredited laboratory. Diagnostic variants were identified in 85 different genes, with diagnoses in 16 genes being causative in more than one unrelated individual.

**Fig. 2:**
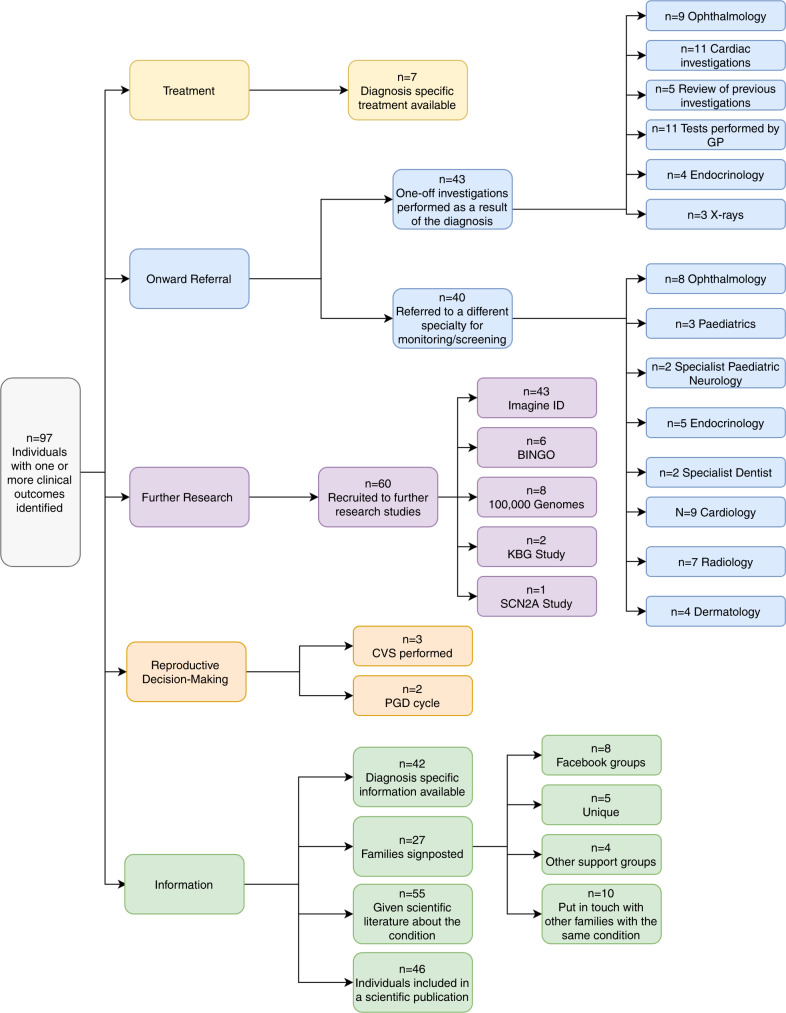
Flowchart demonstrating outcomes identified primarily through the clinical audit. BINGO Brain and Behaviour in Intellectual Disability of known Genetic Origin study, CVS chorionic villus sampling, GP general practitioner, IMAGINE ID Intellectual Disability and Mental Health: Assessing the Genomic Impact on Neurodevelopment study, PGD pre-implantation genetic diagnosis.

Clinical outcomes identified in the audit (or subsequent interview) are summarized in Fig. 2. Seven probands (6%) received a diagnosis for which a disease-specific treatment was known (Table 1). Of these, 5/7 probands had treatable epilepsy as part of their phenotype, where knowledge of the specific molecular cause allows the correct drug to be rapidly selected from those available.^17^ Of the five probands with treatable epilepsy, four received a change in treatment following their genetic diagnosis. One proband received an expedited hematopoietic stem cell transplant to treat bone marrow failure, and another may receive treatment for Parkinsonism when age appropriate.

**Table 1:** Summary of diagnoses where specific treatments were available.

DECIPHER ID	Gene	Treatment	Primary phenotype treated	Uptake	Comment
**275863**	*ATP1A3*	Benzodiazepines, dopamine agonists or levo-dopa	Epilepsy	Y	Treatment changes reported to reduce frequency and severity of seizures
**305456**	*GRIN2B*	N-methyl D-aspartate receptor blockers such as memantine or radiprodil	Epilepsy	N	Patient’s seizures already reasonably controlled with different medication
**282007**	*KCNQ2*	Carbamazepine	Epilepsy	Y	Patient was prescribed carbamazepine, which was reported to reduce seizure frequency
**273914**	*PHGDH*	L-serine	Epilepsy	Y	Treatment did not have a significant impact on patient’s symptoms; patient was aged 4 years when treatment given; highest efficacy rates reported when given in utero
**264089**	*SCN2A*	Lamotrigine	Epilepsy	Y	Treatment changes reported to improve seizure control
**291232**	*LIG4*	Hematopoietic stem cell transplantation	Bone marrow failure	Y	Prophylactic hematopoietic stem cell transplant performed 1.5 years after the diagnosis
**275418**	*WDR45*	Parkinson treatments	Early parkinsonism (teens)	N	Patient was not yet in their teens

A total of 43 further diagnostic clinical investigations and 40 referrals to different medical specialties were requested for 48 individuals (43%). These included requests for reviews of previous scans (*n* = 5), ophthalmology assessments (*n* = 9), and cardiac investigations (*n* = 11), and referrals for ongoing screening by radiology (*n* = 7), ophthalmology (*n* = 8), and endocrinology (*n* = 5) departments. Many of these individuals did not present with problems requiring those medical specialties prediagnosis. Information relating to the results of these screening referrals was not assessed through this audit. Furthermore, we were able to identify two participants for whom the diagnosis prevented unnecessary diagnostic interventions, including growth investigations and a magnetic resonance imaging (MRI) scan under general anesthetic.

A total of 60 families (53%) were recruited to additional research studies following their diagnosis, including the Intellectual Disability and Mental Health: Assessing the Genomic Impact on Neurodevelopment (IMAGINE ID) study^18^ (*n* = 43) and the Brain and Behaviour in Intellectual Disability of known Genetic Origin (BINGO) study^19^ (*n* = 6). Gene or disease-specific information in the form of a patient information leaflet or document was given to 42 families (37.5%). A total of 27 families (24%) were signposted to sources of additional information, including the Unique website (*n* = 5), Facebook groups (*n* = 8), and disease-specific support groups (*n* = 4). Contact between the families of probands with the same diagnosis was facilitated for ten families.

Discussions with parents regarding reproductive choices were documented in the notes of 45 families, which represents 83% of couples where the mother was of reproductive age (<45 years) at the point of receiving the proband’s diagnosis. The audit identified three cases in which the proband’s diagnosis was used to inform invasive prenatal testing procedures in subsequent pregnancies, all of which were negative. In a further two cases, the couples chose to have preimplantation genetic diagnosis; treatment for one couple is still ongoing, and the second couple now have a healthy child. These five families represent 9% of the families in which the mother was of reproductive age.

The audit identified one adverse effect of receiving a diagnosis. The proband showed distress during their results appointment upon learning that the diagnosis would likely lead to additional symptoms and screening in the future, requiring further face-to-face and telephone consultations to manage their distress and anxiety.

**Fig. 3:**
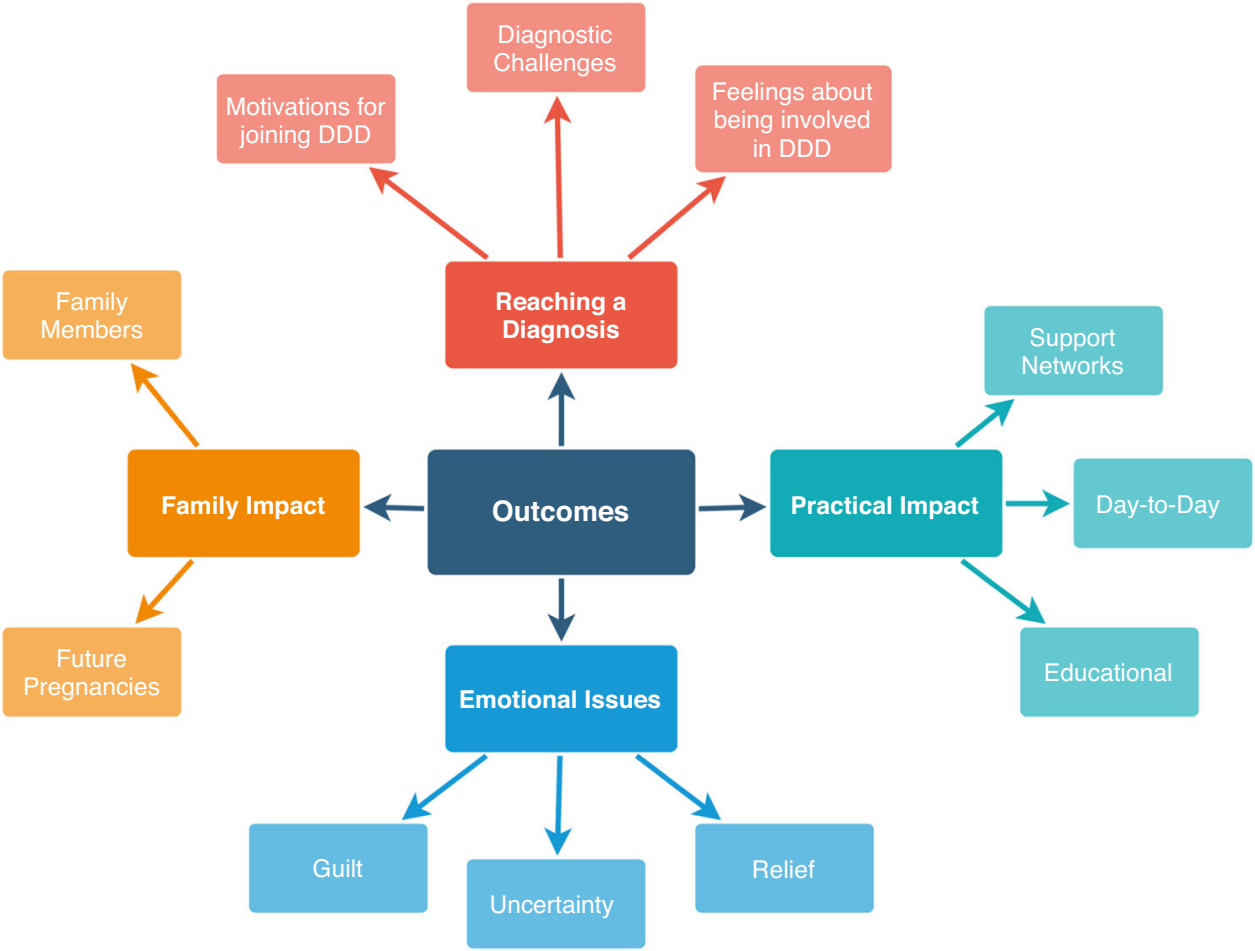
Cluster diagram representing the four major themes and their respective subthemes identified through thematic analysis of the interview transcript data. DDD Deciphering Developmental Disorder study.

We were unable to identify any outcomes beyond the diagnosis itself for 16/112 individuals in the clinical audit (Fig. 1). Of these, one proband was deceased, so 15 families were invited to participate in a telephone interview. Seven initial responses were received (47% response rate) from parents of patients, and six interviews were conducted. The probands in these six families had all received a diagnosis of a de novo variant identified through the DDD study. Thematic analysis identified four major themes and a range of subthemes, shown in Fig. 3, and selected quotes from interview transcripts are given in Supplementary Table 3.

All interview participants were positive about having participated in the DDD study, and one participant described a treatment change that had arisen for their child following the diagnosis that was not identified in the clinical audit (included in Fig. 2 and Table 1). The main motivation for participating in the study was finding an answer for their child’s problems, with many describing a long and complicated search for a diagnosis.^20^ Parents felt a sense of responsibility to obtain a diagnosis, and also described secondary altruistic motivations. Two families described the negative impact of previous incorrect diagnoses, with one interview participant reporting difficulty in accepting a diagnosis that did not seem to fit the child’s problems. Most participants reported feelings of guilt about their child’s difficulties, and upon learning that the genetic variant has arisen de novo, some participants reported that their guilt had reduced. While all participants acknowledged that their child’s diagnosis had provided clarity as to the cause of their symptoms, for some, the diagnosis also brought uncertainty, particularly among parents of younger probands (*n* = 3 with children <15 years).

All participants discussed the impact of their child’s diagnosis on other family members, and valued the relevance of the information for the wider family. Three participants raised the topic of further children and provided different perspectives of the decision-making process faced by families without a diagnosis. One participant described a subsequent pregnancy before the final diagnosis as an extremely challenging time; the lack of clarity about the cause of her child’s symptoms negatively impacting her experience of the subsequent pregnancy. Another participant described how not having a diagnosis previously had impacted on the couple’s decision not to have another child.

Despite feeling generally positive about receiving a diagnosis, all six interviewees explained that the level of day-to-day needs of, and support for, their child had not substantially changed following the diagnosis. Nonetheless, parents did feel that the process of applying for support had been streamlined by having a definitive genetic diagnosis rather than a list of symptoms. One participant described the diagnosis as being key to accessing specialist educational support, which significantly improved the child’s wellbeing. There was disparity among the interview participants about accessing support networks; interestingly, those who were aware of similarly affected children often did not wish to make contact due to fear of overwhelming the other family or because they did not perceive a benefit to such a meeting.

## DISCUSSION

We have evaluated the clinical and psychosocial outcomes in a subset of 112 families from a single UK center who received a genetic diagnosis from the DDD study. We were able to identify diagnosis-specific outcomes in 97/112 (87%) individuals, including direct medical outcomes for the proband in 45% of cases, such as diagnosis-specific treatments (*n* = 7) and referral for additional investigations or screening (*n* = 43). Those patients who received a diagnosis-specific treatment experienced clinical improvements in symptom control, demonstrating the value and importance of tailored treatments,^21,22^ and emphasizing the need for continued research into this field. In contrast to treatment of existing phenotypes, referral for additional screening often related to monitoring and managing phenotypes that had not yet emerged, something that caused anxiety in at least once case.

Some families (*n* = 5) were able to access prenatal testing and preimplantation genetic diagnosis treatments, which would not have been available to them without the diagnosis. We anticipate that many more would have accessed this option had the parents been of reproductive age at the point of receiving the diagnosis. We also expect that this result is likely to have been influenced by the high proportion of families with diagnostic de novo variants in our cohort. Accurate genetic diagnosis of developmental disorders empowers individuals to make informed and value-based reproductive decisions, highlighting the importance of a diagnosis for family planning purposes.^23,24^ For many participants (53%), receiving a diagnosis meant they met the eligibility criteria for further research studies and subsequent successful recruitment suggests that they had a positive perception of genetic research following their involvement in the DDD study. The audit identified 46 participants who had been included in a scientific publication, demonstrating the benefits of novel diagnoses to the scientific community. Additionally, we found that around a third of participants were given patient-focused information related to the diagnosis while others were signposted to sources of additional information; both are ways to reinforce complex genetic information. In 55 cases, where patient-focused information was not available, scientific literature was instead given to the family where appropriate. These findings highlight and support the need for continued development of patient-focused information to be developed as new developmental disorders are identified.

Our semistructured interviews focused on families in whom no clinically relevant outcomes could be identified from the clinical audit. Although this strategy left us with just 16 eligible families, we judged that it would maximize our chances of identifying additional outcomes while biasing the participants toward those who might feel less favorably toward genetics research and who might thus offer a different and useful perspective. Nonetheless, a good response rate of nearly 50% among those invited to interview suggests high engagement and motivation even in these families for whom no clinical outcomes were observed. Moreover, through the course of just six interviews, we identified an additional family in whom diagnosis enabled a specific treatment. This suggests that we underascertained outcomes based on a clinical audit of genetics notes and indicates that further research using extended hospital notes or additional interviews is needed to fully explore outcomes.

All participants reported feeling positively about their involvement in the DDD study, and while recognizing the potential for bias given that these families all received a diagnosis, this finding supports the value for patients and families of being involved in genetics research. Four main themes and further subthemes were identified following thematic analysis, although there is significant overlap between the themes (Fig. 3 and Supplementary Table 3). The observed sense of parental responsibility to find an explanation for their child’s symptoms links to a reported sense of failure when standard clinical pathways fail to find an answer.^25^ Secondary altruistic motivations demonstrate the different expectations families have of participating in genetic research studies.^26^ Experiences shared by interview participants about the diagnostic odyssey emphasized the complex interplay between their child’s phenotypic symptoms, creating an emotional burden.^20^ One family described their experience with earlier misdiagnoses, demonstrating the widespread negative impact on the patient and the wider family of incorrect diagnoses. This further reflects a theme of parental “gut instinct”;^27^ some parents reported feeling strongly that a diagnosis was incorrect, but did not feel qualified to question or challenge the medical opinion,^28^ potentially increasing self-doubt and further delaying a correct diagnosis.^29^

Most interview participants described feeling significant levels of guilt about their child’s problems. For them, finally knowing the underlying cause changed this guilt, demonstrating the value of timely diagnosis.^30,31^ However, this outcome is partly influenced by the fact that all interviewed families had a diagnostic de novo variant; higher levels of parental guilt have been linked to inherited autosomal recessive or X-linked diagnoses in children.^30^ Relief was a frequently reported emotional impact associated with receiving a diagnosis. Participants associated relief with feelings of validation for their child’s difficulties and their own concerns. Relief has also been linked to parental empowerment,^32^ showing the interplay between parental guilt and relief, and highlighting the value of finally obtaining a diagnosis. Concern for other family members also demonstrates that the impact of not having a diagnosis extends beyond the individual patient, and reflects the importance of a family-based approach in managing highly penetrant monogenic conditions.^33^ Being able to accurately clarify and communicate risks to family members was highly valued by the interview participants, with emotional responses reflecting a sense of parental responsibility in providing information for the whole family.^25^ Interview participants differed in their decision to have another child and their uptake of testing during subsequent pregnancies, highlighting the complexities of such decisions. The interviews demonstrate that these emotions can last for a number of years, and can resurface in the future, emphasizing the emotional burden felt by parents without a diagnosis. One participant’s reflection that having an earlier diagnosis might have influenced the decision to have another child emphasizes the challenges faced in reproductive decision-making in a rapidly changing field.

### Limitations

Our findings are limited by a few practicalities. First, although genetic and phenotypic data on DDD families is managed centrally and shared with clinical teams via the DECIPHER database, a linked-anonymized system of IDs is used and researchers do not have access centrally to personal identifiers, which are maintained inside local NHS clinics. We therefore limited our sample group to one UK Regional Genetics Service, so our conclusions may not be representative of different services or geographies. Second, medical records at the Royal Devon and Exeter NHS Foundation Trust are not currently electronic, and clinical notes from different specialties are often held in numerous different locations and institutions across the region. Our clinical audit was limited to notes held within the Peninsula Clinical Genetics Service, so actions undertaken by other specialties may have been missed. Third, we decided to focus our interviews on individuals in whom we were unable to identify any outcomes from the clinical audit, so our sample size for the qualitative phase of the study was small. Additionally, only a small number of interviews were performed, limiting our ability to draw generalizable conclusions. Nonetheless, we were able to develop and apply a systematic approach to evaluating outcomes across an existing research cohort, and future research could utilize the same methodology with larger sample sizes across multiple genetics services (led by individuals embedded within each Regional Genetics Service to access local patient notes), allowing for greater comparative analysis. Additional research could also expand to include information from clinical notes outside of genetics, and potentially also explore similar themes in more participants regardless of whether a clinical outcome had been recorded in the notes.

### Conclusion

Knowledge of the underlying molecular etiology of disease has intrinsic value and is a worthwhile outcome of genetic testing and research.^34,35^ Nonetheless, showing how that knowledge is used and to what, if any, clinical purposes it can be put is of great importance for assessing the wider medical, social, and economic significance of a genetic diagnosis. This study aimed to systematically assess both clinical and nonclinical outcomes associated with receiving a diagnosis through a subset of participants in the DDD study. We show that receiving a diagnosis contributes to clinically relevant outcomes in a high proportion of individuals with developmental disorders and can streamline future clinical care. Although a disease-specific treatment was available to only a small number of individuals at the time of writing, the majority of these treatments were associated with an improvement in symptoms and more targeted treatments are likely to become available in the future. Furthermore, through the interview process, we found that even for those individuals without clinical outcomes, participating in a genetics research study was a positive experience and receiving a diagnosis can have significant personal, emotional, and psychosocial outcomes for the patient and their wider family.

## Supplementary Information

Supplementary Tables 1-3

## Data Availability

Diagnostic variants and phenotypes for probands included in this study are available via the DECIPHER database (https://decipher.sanger.ac.uk/).
