# Supplementary material for: Systematic assessment of outcomes following a genetic diagnosis identified through a large-scale research study into developmental disorders
Source: Genet Med. 2021 Feb 18;23(6):1058–64. doi: 10.1038/s41436-021-01110-3 (PMC8187151; doi:10.1038/s41436-021-01110-3)
Supplement: Supplementary file 1 — Supplementary Tables 1-3 [file 41436_2021_1110_MOESM1_ESM.pdf]

Supplementary Table 1
Pro forma used to identify outcomes through the clinical audit from the genetics notes of participants

General Information	Is the patient alive?
	Genetics ID
	DDD ID
	Gene
	Inheritance Pattern
Treatment / Clinical Changes	Is a diagnosis specific treatment available?
	Was the patient given the treatment?
	Details
	Was the patient referred for any one-off investigations
	Was the patient referred for any ongoing screening?
	Were there any interventions avoided as a result of diagnosis?
Research	Was the patient recruited to a further research study?
Reproductive Choices	Paternal age at time of diagnosis
	Maternal age at time of diagnosis
	Was PND discussed with parents?
	Was PND performed?
	Details
Information	Is diagnosis specific information available?
	Was the family given any scientific literature about the condition?
	Were the family included in a scientific paper?
Adverse Effects	Are there any known adverse effects?

Supplementary Table 2
Semi-structured Interview Questions used to evaluate non-clinical outcomes

1. *What made you want to take part in the DDD study?*
2. *What was your experience of taking part in the DDD study?*
3. *How did it feel to receive the diagnosis for (child's name)?*
4. *Has (child's name)'s diagnosis changed things for (a) the child, (b) you as a couple, (c) you as a family?*
5. *Has the diagnosis changed the support you were able to access?*
6. *Have you found other information about diagnosis or other patients?*
7. *What would you say has been the most positive thing about the study?*
8. *What would you say has been the most negative thing about the study?*
9. *Would you change anything about your decision to be involved in the study?*

Supplementary Table 3

Selected quotes from semi-structured interviews with six DDD parents, demonstrating the identified themes.

**Reaching a Diagnosis**	**Motivations for joining the DDD Study**	*"I think anybody who has a child with additional needs feels a need to know"*<br><br>*"[...] and I would say the added hope was that if someone else had a child with the same problems as [child] then they would have more information earlier"*
	**Diagnostic challenges**	*"Because she's never fit into any specific categories, there was nothing the doctors could put their fingers on [...] as parents it was incredibly frustrating"*<br><br>*"I think having [...] I call it the false diagnosis, I remember sitting there thinking [...] this isn't [child], because I'd obviously researched [condition] and I always felt that [child] didn't fit the picture and I tried desperately to make him fit, like really desperately"*
	**Feelings about involvement in the study**	*"I just find the whole thing so interesting and I'm pleased that we took part"*<br><br>*"It's made a huge difference to me as a mum, me as a parent and me as a person, having that knowledge is a great thing, and it wouldn't have happened without the project"*
**Practical Impact**	**Day-to-day support**	*"[...] it has always been a point of frustration for me that I have needed to write [child's] symptoms in a long list over and over again, and so it feels nice to just write the diagnosis and be done with it"*
	**Support networks**	*"[child] has always been compared to normal children her whole life [..] it would be nice to be able to compare her to others with the same situation for a change"*<br><br>*"The helpful bit is knowing the information, it's action rather than talking about it"*
	**Educational support**	*"She was put into mainstream secondary school [...] that first year, it destroyed her, it absolutely destroyed her [...] the diagnosis came in and now she's in a special school [...] she's now a lot happier, she's back on track, she's flying so I can't be happier [...] if the diagnosis hadn't come through I'm honestly not sure of where she'd be"*
**Emotional Issues**	**Guilt**	*"I think it would be true to say that any parent, particularly a mother [...] has a massive burden of guilt [...] it seemed impossible to get away from the sense that I could have done something more about it, done less [...]. It's really such a life-changing feeling to know that nothing I did or could have done has affected the way she is"*
	**Relief**	*"It helped [child] a huge amount because it's given her credibility, and that, for me as her dad, is a huge relief"*<br><br>*"I don't think either of us would have blamed the other one [...] it was just a relief to know that we hadn't affected her life in any way, because it's always in the back of your mind"*

	**Uncertainty**	*"There seems to be a limited amount of information about [condition] life expectancy for example [...] which is always a massive concern to parents, you know, are they likely to outlive us?"*<br><br>*"[...] we're very much aware that [child] has an uncertain future, [...] the diagnosis can't predict his future so there is no path to follow [...] I think we've addressed those issues about his uncertain future already"*
**Family Impact**	**Family Members**	*"[...] at least now I can turn around to my girls and say it's not hereditary [...] it doesn't mean that your children are going to be disabled [...] I can't describe how valuable that feels"*
	**Future Pregnancies**	*"It was a very worrying time for me, I had all of the tests and everything and they all came back fine [...] but the worry was still there, and I honestly didn't enjoy the pregnancy. It felt like a cycle of being anxious and stressed, but then feeling guilty because I knew stress could cause problems and that cycle didn't really go away [...] I had buried those feelings over the years [...] I only really remembered when my son's wife was pregnant and it was like history repeating itself again"*
